# Stomata and Sporophytes of the Model Moss *Physcomitrium patens*

**DOI:** 10.3389/fpls.2020.00643

**Published:** 2020-05-25

**Authors:** Robert S. Caine, Caspar C. C. Chater, Andrew J. Fleming, Julie E. Gray

**Affiliations:** ^1^Department of Molecular Biology and Biotechnology, University of Sheffield, Sheffield, United Kingdom; ^2^Department of Animal and Plant Sciences, University of Sheffield, Sheffield, United Kingdom

**Keywords:** stomatal development, guard cells, guard mother cell, moss, *Physcomitrella*, stomatal function, evolution

## Abstract

Mosses are an ancient land plant lineage and are therefore important in studying the evolution of plant developmental processes. Here, we describe stomatal development in the model moss species *Physcomitrium patens* (previously known as *Physcomitrella patens*) over the duration of sporophyte development. We dissect the molecular mechanisms guiding cell division and fate and highlight how stomatal function might vary under different environmental conditions. In contrast to the asymmetric entry divisions described in *Arabidopsis thaliana*, moss protodermal cells can enter the stomatal lineage directly by expanding into an oval shaped guard mother cell (GMC). We observed that when two early stage *P. patens* GMCs form adjacently, a spacing division can occur, leading to separation of the GMCs by an intervening epidermal spacer cell. We investigated whether orthologs of Arabidopsis stomatal development regulators are required for this spacing division. Our results indicated that bHLH transcription factors PpSMF1 and PpSCRM1 are required for GMC formation. Moreover, the ligand and receptor components PpEPF1 and PpTMM are also required for orientating cell divisions and preventing single or clustered early GMCs from developing adjacent to one another. The identification of GMC spacing divisions in *P. patens* raises the possibility that the ability to space stomatal lineage cells could have evolved before mosses diverged from the ancestral lineage. This would have enabled plants to integrate stomatal development with sporophyte growth and could underpin the adoption of multiple bHLH transcription factors and EPF ligands to more precisely control stomatal patterning in later diverging plant lineages. We also observed that when *P. patens* sporophyte capsules mature in wet conditions, stomata are typically plugged whereas under drier conditions this is not the case; instead, mucilage drying leads to hollow sub-stomatal cavities. This appears to aid capsule drying and provides further evidence for early land plant stomata contributing to capsule rupture and spore release.

## Introduction

Stomata are microscopic pores typically consisting of a pair of guard cells which regulate a central aperture to control gas exchange for photosynthesis and water loss. They are present on the majority of land plants and evolved prior to 418 million years ago (Edwards et al., 1998; Ligrone et al., 2012; Chater et al., 2013, 2017). Along with other structural innovations such as leaves, roots and a cuticle, stomata permitted plants to grow larger and thrive in drier environments; which in-turn resulted in vegetation increasingly impacting on, and shaping the terrestrial biosphere (Beerling, 2007; Berry et al., 2010). Vascular land plant stomata regulate plant gaseous exchange, water status, temperature, internal solute transport and can also prevent or allow pathogen entry (Yoo et al., 2010; Franks et al., 2015; Hepworth et al., 2015; Dutton et al., 2019). The role of stomata in non-vascular land plants is less well understood, but recent evidence points toward a role in aiding sporophyte capsule drying and spore release; possibly by facilitating the drying and recession of internal mucilage but does not preclude additional roles (Duckett et al., 2009; Pressel et al., 2014; Villarreal and Renzaglia, 2015; Chater et al., 2016; Duckett and Pressel, 2018). Here, we assess stomatal formation during the sporophyte development of *Physcomitrium patens*, and provide novel insights into how moss stomata develop and function. For a more specialized overview of *P. patens* sporophyte development, see Hiss et al. (2017).

**FIGURE 1:**
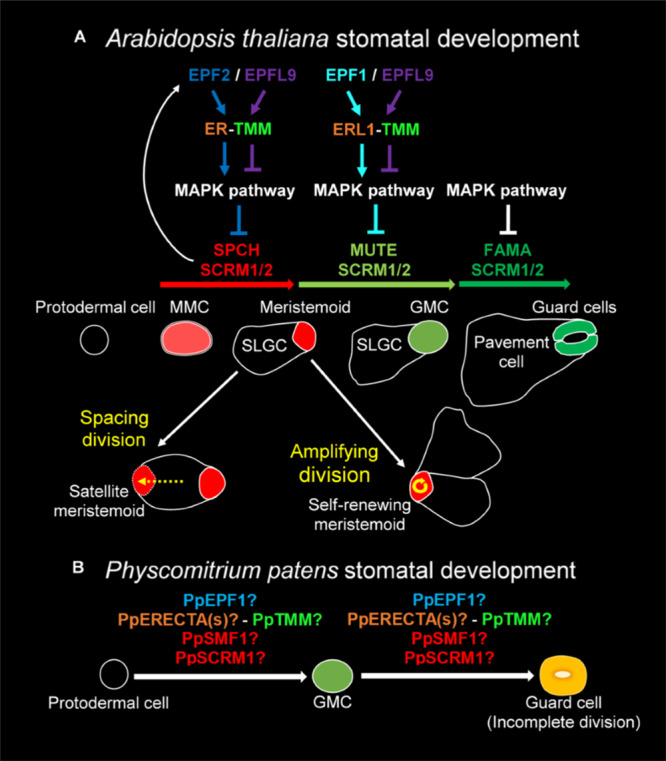
Overview of the molecular control of stomatal development. **(A)** In *Arabidopsis thaliana* the stomatal lineage is initiated when an undifferentiated protodermal cell is specified to become a meristemoid mother cell (MMC) via the actions of a heterodimeric protein complex consisting of bHLH transcription factors SPEECHLESS (SPCH) and either SCREAM (SCRM) or SCRM2 (also referred to as ICE1 and ICE2). The MMC then undergoes an asymmetric entry division again promoted by SPCH and SCRM/2 activity. SPCH is phospho-regulated via the activity of a mitogen activated protein kinase (MAPK) pathway that facilitates signals being relayed from the plasma membrane to the nuclear-residing transcription factors. TOO MANY MOUTHS (TMM) and ERECTA family proteins act to regulate external signals that trigger the MAPK pathway from outside the cell. Binding of EPIDERMAL PATTERNING FACTOR (EPF) 2 signaling peptide to ERECTA family proteins particularly ERECTA (ER) increases MAPK activity and SPCH is phosphorylated thereby preventing stomatal lineage progression. Conversely, if EPF-like 9 (EPFL 9, also known as STOMAGEN) outcompetes EPF2, then the MAPK pathway is not activated and SPCH activity is preserved. The heterodimeric unit of SPCH and SCRM/2 is self-regulatory as SPCH drives the expression of EPF2. Additional meristemoids can be generated via spacing divisions where an SLGC divides away from an already formed meristemoid, and via self-renewing amplifying divisions of an existing meristemoid. Both of these divisions are solicited by SPCH and SCRM/2 activity. For meristemoids to advance to guard mother cell (GMC) state, the bHLH MUTE is required in combination with SCRM/2. This is again under the control of the MAPK pathway. Instead of EPF2, EPF1 competes with EPFL9 for the binding of ERECTA proteins particularly ER-like 1 (ERL1) with moderation again via TMM. EPF1 bound ERL1 leads to increased MAPK activity and prevents stomatal lineage advancement. For a GMC to symmetrically divide and form a pair of guard cells (GCs) both MUTE, and then FAMA, again in combination with SCRM/2 are required. FAMA, like SPCH and MUTE, is also regulated via MAPK activity, although how this occurs is not well understood. **(B)** A suggested model for *Physcomitrium patens* stomatal development. Mosses and hornworts initiate stomatal development module when a protodermal cell expands and becomes an ovoid GMC. For *P. patens*, the GMC then undergoes an incomplete symmetric division leading to the formation of a single ovoid shaped GC. Where the previously described *PpSMF1*, *PpSCRM1, PpTMM, PpEPF1*, and *PpERECTA1* gene products function during the developmental module remain to be determined.

The development of stomata in vascular land plants is well described, with *Arabidopsis thaliana* being particularly well characterized (Zhao and Sack, 1999; Lucas et al., 2006; Rudall et al., 2013, 2017; Figure 1A). Recent work in mosses and hornworts has begun to dissect the relatively simpler mechanisms of stomatal development in bryophyte species (Merced and Renzaglia, 2013, 2016, 2017; Pressel et al., 2014). Like other mosses, the model species *P. patens* (Rensing et al., 2020) employs a simple form of stomatal development (Figure 1B) and a number of genes orthologous to those of Arabidopsis have been shown to regulate stomatal development and patterning (MacAlister and Bergmann, 2011; Caine et al., 2016; Chater et al., 2016). Surprisingly, despite the elucidation of genetic regulators, our understanding of stomatal and epidermal ontogeny in this model moss remains limited (Figure 1B). This represents a significant gap in our knowledge relating to how stomatal development might have altered over the course of evolution. With a better understanding of how *P. patens* produces stomata, we will gain insight into how stomatal and epidermal cell coordination has developed over time, and may begin to understand how vascular land plants gradually built the intricate stomatal developmental and patterning modules that we marvel at today.

Most of our knowledge relating to the genetics underpinning stomatal development stems from work conducted on Arabidopsis (Qi and Torii, 2018; Zoulias et al., 2018; Lee and Bergmann, 2019). Research presented here is associated with the core signaling module regulating stomatal development, outlined in Figure 1A, but for an in-depth description relating to the latest findings see also Lee and Bergmann (2019) and Qi and Torii (2018). Arabidopsis stomatal development is initiated when basic helix-loop-helix (bHLH) transcription factors SPEECHLESS (SPCH) and ICE1/SCREAM (SCRM) or ICE2 (SCRM2) heterodimerize leading to a subset of protodermal cells gaining meristemoid mother cell (MMC) identity (Figure 1A; MacAlister et al., 2007; Kanaoka et al., 2008). MMCs undergo an asymmetric entry division to produce a smaller meristemoid cell and a larger stomatal lineage ground cell (SLGC), again regulated via SPCH-SCRM/2 (Zhao and Sack, 1999; Bhave et al., 2009; Pillitteri and Dong, 2013). SLGCs either produce satellite meristemoids via an asymmetric spacing division (regulated by SPCH-SCRM/2 activity), or can de-differentiate and form epidermal pavement cells (Pillitteri and Dong, 2013). The meristemoids formed by either entry or spacing divisions may also undergo self-renewing amplifying divisions, which leads to the production of further SLGCs, again via SPCH-SCRM/2 activity (Zhao and Sack, 1999; Berger and Altmann, 2000; MacAlister et al., 2007; Kanaoka et al., 2008).

Following these asymmetric divisions, the transition from Arabidopsis meristemoid to guard mother cell (GMC) is orchestrated by the transcriptional regulator bHLH MUTE (closely related to SPCH), in combination with SCRM/2 (Figure 1A; Kanaoka et al., 2008; Pillitteri et al., 2008). To form a pair of guard cells, MUTE and SCRM/2 also oversee the GMC symmetric division, and finally FAMA (related to both SPCH and MUTE), together with SCRM/2, enforces correct guard cell identity (Zhao and Sack, 1999; Ohashi-Ito and Bergmann, 2006; Kanaoka et al., 2008; Han et al., 2018). The activity of SPCH, MUTE and FAMA, in combination with either of the SCRMs, is modulated by a mitogen activated protein kinase (MAPK) pathway (Lampard, 2009; Han and Torii, 2016; Qi et al., 2017). This pathway facilitates intricate signaling within and between cells by connecting the plasma membrane to the nuclear bHLH transcription factors (Qi and Torii, 2018; Lee and Bergmann, 2019).

To coordinate the above cellular transitions and divisions, Arabidopsis uses a signaling network which includes a plasma membrane-localized receptor-like protein (RLP), receptor-like kinases (RLKs) and apoplastic signaling peptides (Figure 1A; Qi and Torii, 2018; Lee and Bergmann, 2019). For asymmetric divisions to commence, the RLP TOO MANY MOUTHS (TMM) in combination with the ERECTA family of RLKs [especially ERECTA (ER)] are particularly important (Yang and Sack, 1995; Shpak et al., 2005; Lee et al., 2012, 2015). These receptor components enable the transduction of signals from the apoplast across the plasma membrane. If the apoplastic signaling peptide EPIDERMAL PATTERNING FACTOR (EPF) 2 is successful in binding to TMM and ERECTAs (typically ER) then asymmetric entry divisions fail to occur and the stomatal lineage is halted because SPCH becomes phosphorylated and thus de-activated (Figure 1A; Hara et al., 2009; Hunt and Gray, 2009; Lee et al., 2015). Conversely, If EPF2 is out-competed for receptor binding by EPF-like 9 (EPFL9, otherwise known as STOMAGEN), then an asymmetric entry division is more likely to occur, resulting in a meristemoid and SLGC (Hunt et al., 2010; Sugano et al., 2010; Lee et al., 2015). The subsequent transition of the meristemoid to a GMC is governed by EPF1, which like EPF2, uses TMM and ERECTA family members [particularly ER-like1 (ERL1)] to convey signals into the cytoplasm that prevent GMC formation (Hara et al., 2007; Hunt and Gray, 2009; Lee et al., 2012; Pillitteri and Torii, 2012; Qi et al., 2017).

We have previously shown that *P. patens* expresses multiple genes that are orthologous to Arabidopsis equivalents that function during stomatal development and patterning (Caine et al., 2016; Chater et al., 2016). Instead of three bHLH genes akin to *SPCH*, *MUTE* and *FAMA*, *P. patens* has two genes: *PpSMF1* and *PpSMF2*, of which only *PpSMF1* is required during stomatal development. In *ppsmf1* knockout lines no stomata form on the moss sporophyte (Chater et al., 2016). For Arabidopsis SCRM/2 equivalents, there are four moss orthologs, of which, only *PpSCRM1* has thus far been identified to be involved in stomatal development. As with *ppsmf1* plants, *ppscrm1* mutants possess no stomata on the moss sporophyte (Chater et al., 2016). Whilst Arabidopsis has three *ERECTA* family genes, there are six orthologous *P. patens* genes, but to date only *PpERECTA1* has been studied (Caine et al., 2016). Like ERECTA, PpERECTA1 positively regulates stomatal development and the correct placement of stomata. The contribution of other *PpERECTA* genes to stomatal and sporophyte development remains unknown.

TOO MANY MOUTHS is a single-copy gene in both Arabidopsis and *P. patens* (Caine et al., 2016). Arabidopsis *TMM* prevents stomatal clustering in a number of organs including leaves, yet at the same time promotes stomatal development in other organs, most notably on siliques and the base of inflorescence stems (Geisler et al., 1998; Abrash and Bergmann, 2010). In *pptmm* mutants, clustered stomata and zones devoid of stomata can be found on the same sporophyte, highlighting the complex mechanisms by which PpTMM titrates different stomatal developmental signals during sporophyte growth (Caine et al., 2016). Whilst at least three *EPF/L* genes regulate Arabidopsis stomatal development, only one *EPF* appears to play a role in moss stomatal development: *PpEPF1* (Chater et al., 2017). PpEPF1 negatively regulates stomatal development as *ppepf1* mutants exhibit abnormal contiguous clustering of stomata (Caine et al., 2016). To test whether a positive regulator of stomatal development could promote stomatal development in *P. patens*, Arabidopsis *EPFL9* was over-expressed, but no change in phenotype was detected (Caine et al., 2016). Based on this and phylogenetic analysis, it is likely that positive regulation of stomatal development by *EPFL9* genes evolved after the divergence of vascular plants (Chater et al., 2017). Despite these advances, the coordinated functioning of signaling components in moss stomatal development is yet to be described.

In most non-vascular land plants, stomatal development involves the specification of a protodermal cell which enlarges to become a GMC, that subsequently divides to produce a pair of guard cells (Vaten and Bergmann, 2012; Merced and Renzaglia, 2016, 2017; Figure 1B). GMCs have previously been termed “guard cell parent cells” (GPCs) in mosses and “stomatal mother cells” (SMC) in hornworts (Sack and Paolillo, 1985; Pressel et al., 2014). For simplicity, and to convey their similar shared identity, we refer to all bryophyte equivalents as GMCs. No asymmetric entry division appears to precede GMC formation in non-vascular plants (Rudall et al., 2013; Pressel et al., 2014; Merced and Renzaglia, 2016). Moreover, amplifying divisions and spacing divisions are also thought to be absent in mosses and hornworts. This implies that non-vascular land plant stomata are perigenous as they develop – that is, there is an absence of any neighboring cells that originally derived from the same stomatal lineage (Rudall et al., 2013). GMC development and division in mosses and hornworts appear to be intricately coordinated with chloroplast behavior, as specific chloroplast conformations have been observed prior to the symmetric division that forms the pore (Pressel et al., 2014; Merced and Renzaglia, 2016, 2017). Whilst most bryophyte GMCs divide to produce two guard cells, in the Funariaceae mosses such as *P. patens* the GMC does not fully divide and a single guard celled stomate is produced (Figure 1B; Sack and Paolillo, 1985; Field et al., 2015; Chater et al., 2016).

Most mosses and hornworts, but not liverworts, possess stomata on their sporophytes (Chater et al., 2017; Merced and Renzaglia, 2017; Duckett and Pressel, 2018; Brodribb et al., 2020). For a number of bryophyte species (including *P. patens*), liquid mucilage is initially detectable in the cavity formed beneath the developing stomata of young sporophytes (Pressel et al., 2014; Merced and Renzaglia, 2016; Renzaglia et al., 2017). As sporophyte expansion continues, stomatal opening occurs and typically internal mucilage recedes. This leads to a hollowing of the sub-stomatal cavity. In hornworts this is followed by stomata collapsing inwardly and cells dying as capsules mature (Renzaglia et al., 2017). Mucilage recession enables water release from both moss and hornwort capsules, and this appears to accelerate sporophyte drying and capsule rupture (Villarreal and Renzaglia, 2015; Chater et al., 2016; Merced and Renzaglia, 2017; Duckett and Pressel, 2018). Despite this, observations of near-mature *P. patens* capsules show that sub-stomatal cavity mucilage does not always recede (Chater et al., 2016), suggesting that water release during maturation is variable and possibly conditional on the surrounding environment (Merced and Renzaglia, 2017).

Based on the findings reported here, we suggest that stomatal development in moss is more complex than previously thought and is not exclusively perigenous. We observed mesoperigenous development, with non-stomatal lineage cells forming during the development of stomata (Payne, 1979; Rudall et al., 2013). Furthermore, we dissect how *P. patens* correctly initiates stomatal development and patterning in relation to GMC formation by following deviations of cell fate transitions in *ppsmf1*, *ppscrm1*, *pptmm*, and *ppepf1* knock-out mutants. By analyzing the development and maturation of guard cells and subtending cavities under differing environmental conditions, we provide further insight into the possible role of moss stomata and provide a rationale for whether stomata remain open or become plugged as sporophyte capsules mature.

## Materials and Methods

### Plant Materials

**FIGURE 2:**
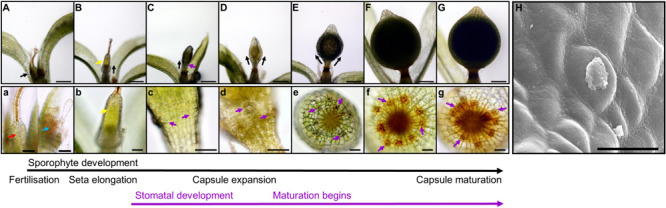
Stomatal development during sporophyte development in *Physcomitrium patens*. **(A–G)** Overview of the developing *P. patens* sporophyte from fertilization to fully expanded brown sporophyte stage. **(a–g)** Close-ups of **(A–G)** illustrating early sporophyte development, and then once formed, stomata and their development in relation to overall sporophyte development. Representative stomata in panels **(c–g)** are marked with purple arrows. **(A)** Mature gametangia and nascent sporophyte (black arrow) surrounded by leafy gametophyte tissue. **(a)** Left image, a very young sporophyte (red arrow) resulting from the fertilization of the egg cell in the female archegonia. The gametophytic calyptra derived from the archegonia is visible and is being pushed up by the underlying nascent sporophyte. Right image, male antheridia (blue arrow) with a cloud of spermatozoids above. **(B)** Developing sporophyte (black arrow) being pushed up via a seta, with gametophyte calyptra still affixed (yellow arrow); the seta is subtending the calyptra. **(b)** A close-up of the calyptra sitting atop the gametophyte (yellow arrow). **(C)** Elongating sporophyte with a darkened central spore sac becoming visible. **(c)** Stomatal lineage cells protruding from the surface of epidermis (purple arrows). The calyptra is absent from this image, and also for subsequent images through to sporophyte maturity. Normally it remains present until the penultimate stages of sporophyte development when sporophytes remain undisturbed (Hiss et al., 2017). **(D)** As the sporophyte begins to expand outward the central spore sac becomes distinct from the surrounding tissue. **(d)** As expansion of the capsule occurs **(D)** stomatal pores can be seen in the central regions of recently formed guard cells (GCs; see centrally placed purple arrow). **(E)** As the central spore sac expands the overall shape of the sporophyte becomes more spherical. **(e)** The stomata on the expanding sporophyte begin a transition from being translucent to being filled with an orange to brown substance. **(F)** A fully expanded green sporophyte with maturing spores. **(f)** The GCs are now orange in color as the sporophyte is maturing. **(G)** The fully expanded sporophyte capsule is browned, indicating that the internal spores are mature. **(g)** Like the sporophyte capsule, the color of the stomata turns increasingly brown prior to and during senescence. **(H)** Scanning electron microscopy image of a mature *Physcomitrium patens* guard cell plugged with waxes. Scale bars are as follows: **(A–G)** = 100 μm; **(a–g)** = 50 μm; **(H)** = 25 μm.

**FIGURE 3:**
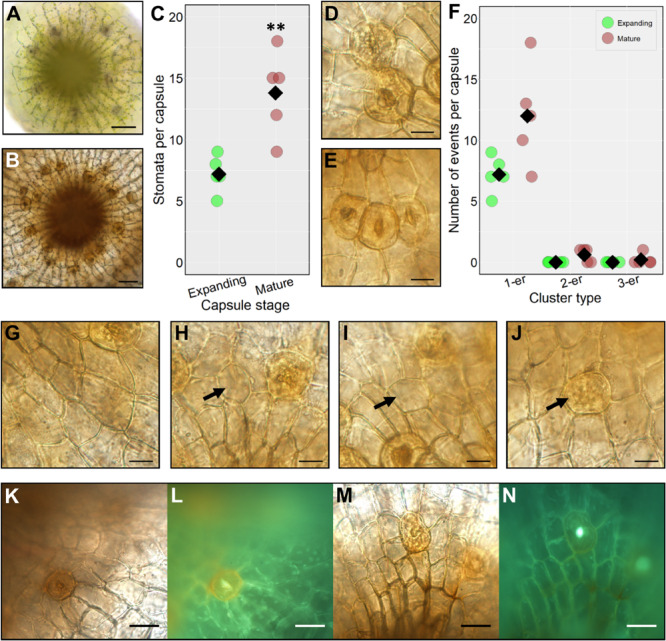
Stomatal formation during capsule expansion and in fully expanded mature capsules. **(A)** A stacked and flattened image of an early expanding sporophyte capsule dissected and face-up. The capsules surveyed were equivalent in size to sporophytes including and between growth stages in Figures 2D,E. **(B)** A stacked and flattened image of an equivalent fully expanded mature brown spore capsule equivalent to Figure 2G. **(C)** Dot-plot of stomatal number on expanding and mature sporophyte capsules. Individual replicate values denoted by circles, means by black diamonds. A two-tailed *t*-test confirms significantly more stomata on mature brown capsules (^∗∗^*P* < 0.01). **(D)** A stomatal cluster consisting of two stomata (2-er) on a mature browned wild-type sporophyte capsule. **(E)** A stomatal cluster consisting of three stomata (3-er) on a mature wild-type capsule. **(F)** Dot-plot of instances of clustering on expanding and mature brown sporophyte capsules. Symbols are as in panel **(C)**. Non-clustering stomata are counted as 1-ers. **(G–N)** All images taken from mature brown fixed capsules. **(G)** An epidermal area almost devoid of stomata. **(H,I)** Enlarged round, possibly aborted GMC cells (see black arrows). **(J)** An aborted GMC displaying the characteristic orange to brown hue akin to mature guard cells but without the central pore (see arrow). **(K)** Mature sporophyte capsule dissected and stained with diphenylboric acid 2-amino ethyl ester (DPBA). **(L)** Capsule exposed to UV light to assess flavonoid derivatives which are visible as an orange fluorescence in the guard cell. **(M)** Mock treated equivalent to panel **(K)**. **(N)** No fluorescence is emitted from the guard cell under equivalent UV light treatment. Scales bars are as follows: **(A,B)** = 50 μm; **(D,E)** and **(G–J)** = 15 μm; **(K–M)** = 25 μm.

**FIGURE 4:**
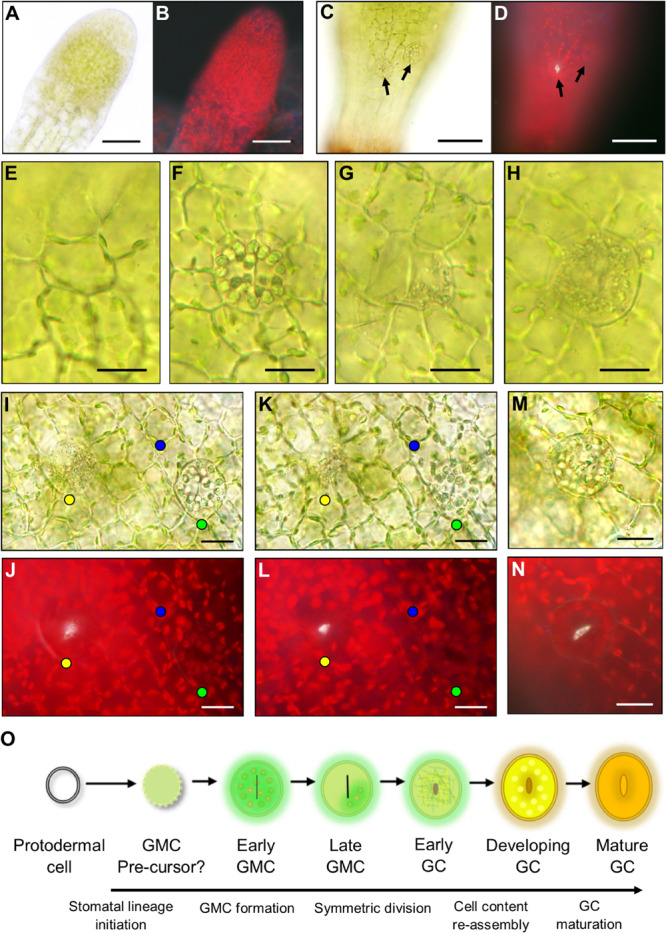
Stomatal lineage cells during stomatal development in *Physcomitrium patens*. **(A)** An excised apical portion of an expanding sporophyte, with calyptra removed, equivalent in size to the sporophyte in Figure 2B. No stomata were visible at this developmental stage. **(B)** Fluorescence image of panel **(A)**. **(C)** Representative elongating sporophyte capsule beginning to expand which is equivalent to a sporophyte between stages Figures 2C,D. A nascent guard cell (GC) can be seen in the central basal region of the sporophyte capsule. More apically and to the right a guard mother cell (GMC) is also present. Note: capsules equivalent to Figure 2C were also used during these observations and nascent stomata were found at this development stage. **(D)** Fluorescent image of the same sporophyte as **(C)** emitting a white autofluorescence coming from the open central open pore of the nascent GC, but no fluorescence was detected from the GMC located more apically and to the right. **(E–L)** Images taken from sporophytes equivalent in size to Figure 2C through to Figure 2D where sporophytes were still elongating and beginning to expand. In most cases a neatly arranged group of cells exists around the central stomatal lineage cells. **(E)** A smaller circular cell which is probably a GMC pre-cursor. **(F)** An expanded GMC with organelles radially aligned at the cell perimeter and a central cell plate. **(G)** Expanded GMC with partially fragmented organelles in one location that are dissipating and a very pronounced cell plate. **(H)** An early GC with fragmented organelles and a central indented region. **(I)** Bright-field image of an early GC with fragmented organelles (yellow dot). A GMC with aggregated organelles (green dot) and a circular cell with organelles circulating around the cell perimeter (blue dot). **(J)** Equivalent fluorescence image to bright-field in panel **(I)**. **(K,L)** Adjusted depth of field images equivalent to those in panesl **(I,J)**, illustrating a fluorescent material in the pores of the GMC with fragmenting organelles (yellow dot), but not in the cell with aggregating organelles (green dot) or the smaller circular undifferentiated cell (blue dot). The fluorescent material is used as a marker for pore formation in the early GC with fragmenting organelles, which is not present in the aggregating organelle GMC or the smaller circular cell. **(M)** Once the organelles have finished fragmenting the pore is formed leading to the reformation of aggregated organelles in the developing GC and a yellow to orange hue beginning to occur inside the developing GC. **(N)** Fluorescence image of panel **(M)** illustrating fluorescence material lining the pore lips. **(O)** Schematic representation of the transition from protodermal cell to Mature GC. Firstly, protodermal cells marginally expand and become surrounded in a very particular cellular arrangement thereby probably becoming a GMC pre-cursor. Then this cell expands further to become early GMC which has aggregated organelles and a central cell plate. As the symmetric division begins to occur, the organelles then dissipate and the late GMC is formed. In early GCs Organelles are fragmented throughout as the central pore begins to form. Organelles then reform in the developing GCs before finally maturing, the process becoming an orangey brown color. Scales bars are as follows: **(A–D)** = 50 μm; **(E–N)** = 15 μm.

**FIGURE 5:**
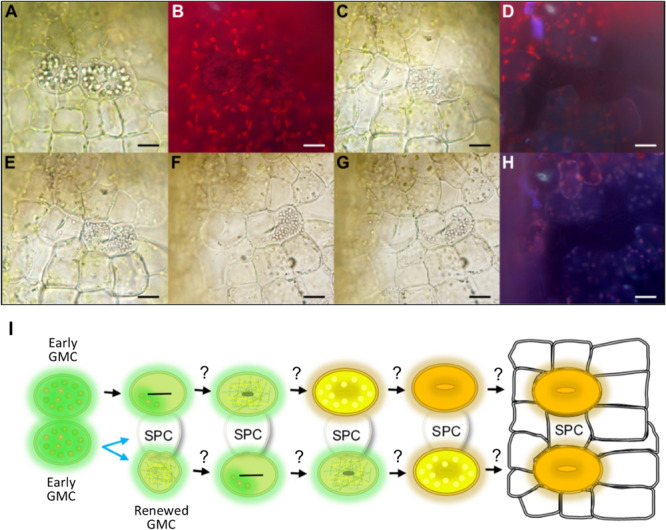
Evidence for mesoperigenous stomatal development in *Physcomitrium patens*. A montage of images taken over approximately 2 h illustrating changes in stomatal lineage cell conformation during an asymmetric GMC spacing division. **(A)** Bright-field image illustrating two neighboring early GMC cells with aggregated cellular organelles. The right GMC is budding off and undergoing an asymmetric spacing division. A cell plate can be seen in both GMCs. **(B)** Fluorescence image of panel **(A)** with no visible build-up of fluorescence material in central pore regions of either cell, further suggesting that both cells are early GMCs (see also Figure 4). **(C)** The previously pronounced organelles in both GMC cells have dissipated substantially in the left-side GMC, and have fragmented in an equally distributed pattern within the right-side cell. **(D)** Fluorescence image of panel **(C)** still displaying no evidence of fluorescent material associated with pore formation. A number of the cells including the GMCs now appear darker than previously observed in panel **(B)**. **(E–G)** Over time as cell division is continuing, the fragmented organelles seem to migrate past the cell plate and become concentrated in the right of two newly forming cells. This leads to the formation of a spacer cell (SPC) in between the two previously adjacent early GMCs. **(H)** Fluorescence image of panel **(G)** still with no visible fluorescence build-up in either the left-side GMC or either of the two daughter cells formed from the spacing division of the right-side GMC parental cell. **(I)** Schematic representation of a GMC spacing division. When two early GMCs form next to each other, one early GMC will bud off and undergo an asymmetric spacing division whilst the other appears to approach mid GMC phase (see also Figure 3). The early GMC that buds off may or may not be able to renew early GMC identity. With an SPC in place between stomatal lineage cells, stomatal development may continue as in Figure 3. Scale bars = 15 μm.

**FIGURE 6:**
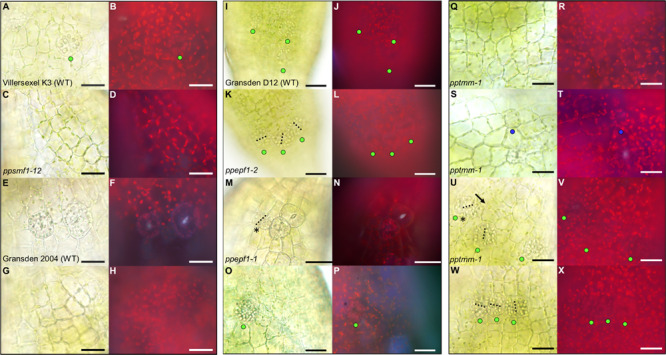
Stomata development-associated genes *PpSMF1*, *PpSCRM1*, *PpEPF1* and *PpTMM* all regulate guard mother cell (GMC) activity. All images displayed are taken from elongating early expanding sporophytes equivalent to those pictured in Figures 1C,D. **(A)** Close-up of Villersexel K3 wild-type (WT) image of the epidermis with a GMC that has yet to undergo division. **(B)** Fluorescent equivalent to panel **(A)**. **(C)** *ppsmf1* epidermis with epidermal cells loosely arranged in files equivalent to wild-type in panel **(A)**. **(D)** Fluorescent image of panel **(C)**. **(E)** Representative close-up Gransden 2004 wild-type (WT) image with nascent guard cells (GCs) that have re-aggregated organelles. **(F)** Fluorescent equivalent to panel **(E)**. Note the fluorescence being emitted from the pore region of newly formed GCs where organelles have re-aggregated. In both wild types a range of different stomatal lineage cell types were detected from early GMCs to nascent GCs at the early expanding sporophyte stage. **(G)** *ppscrm1* epidermis with epidermal cells, but no GMCs, which was equivalent to wild-type in panel **(E)**. **(H)** Fluorescent equivalent to panel **(G)**. **(I)** Gransden D12 wild-type (WT) displaying early GMC cells with accompanying **(J)** fluorescence shot. **(K–X)** All images from plants in Gransden D12 background. **(K)** *ppepf1* with three early GMC cells clustering adjacently with accompanying fluorescence image **(L)**. **(M)** Bright-field and **(N)** fluorescence epidermal images showing ppepf1 nascent early GC and adjacently clustering GMCs. **(O)** *PpEPF1OE* bright-field and **(P)** fluorescence epidermal images of an expanded early GMC that may have undergone- or is in the process of- endoreduplication. **(Q–X)** Bright-field and fluorescence images of *pptmm* sporophyte epidermis’s showing **(Q,R)** undifferentiated cells, **(S,T)** a putative early GMC pre-cursor, **(U,V)** an early GMC dividing toward (see asterisk) another GMC and (W+X) early GMCs clustering. Blue dots represent a probable GMC pre-cursor, green dots represent early GMCs and the yellow dots represent dividing GMCs. Black dotted lines denote cell plates. Scale bars = 25 μm.

**FIGURE 7:**
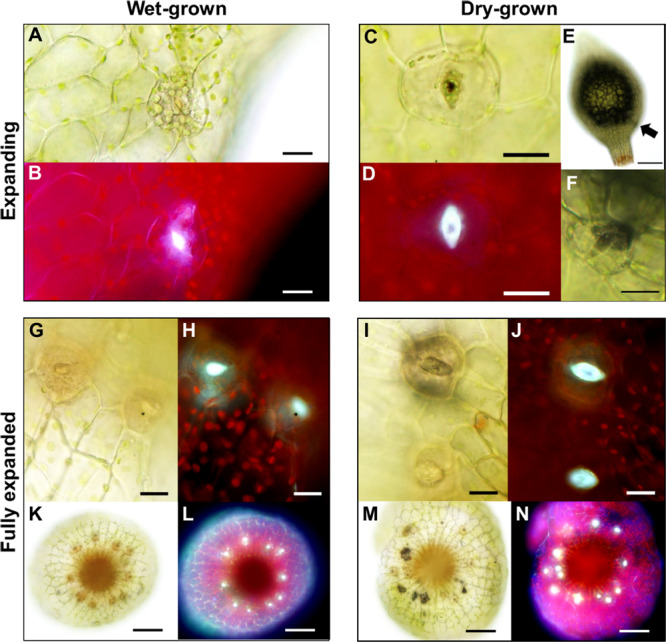
Altered anatomical stomatal functioning in response to different environmental surroundings in *Physcomitrium patens*. **(A)** Bright-field and **(B)** fluorescence images of a nascent guard cell (GC) on an expanding sporophyte growing in wet conditions. Note. The occluded pore. **(C)** Equivalent bright-field and **(D)** fluorescence images of a GC on an expanding sporophyte growing in dry conditions. The differences in fluorescence intensity between panels **(B)** and **(D)** probably represent a difference in mucilage localization with **(D)** exhibiting receding mucilage. **(E)** An expanding sporophyte capsule with a darkened sub-epidermal region with **(F)** corresponding stomata subsequently imaged close up. **(G–J)** Close-up bright-field and fluorescence images of expanded capsules grown under wet **(G,H)** or dry **(I,J)** conditions. Note: in panels **(I,J)** the darkened sub-stomatal cavity and un-occluded pore. **(K–N)** Stacked and flattened images of capsules used to image the stomata in panels **(G–J)**. Note: the darkened cavities and increased fluorescence in **(M,N)** comparatively to **(K,L)**. Scales bars are as follows: **(A–D,F–J)** = 15 μm; **(E,K–N)** = 100 μm.

**FIGURE 8:**
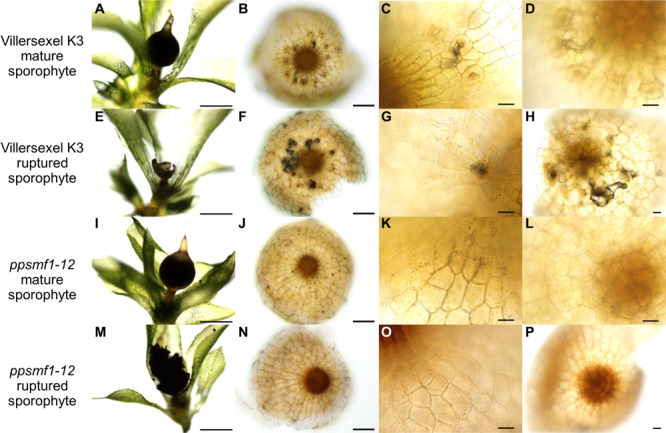
*ppsmf1* moss sporophytes do not possess darkened and vacuous sub-stomatal cavities when grown under dry conditions. For **(A–P)** plants and corresponding sporophytes were taken from plates grown under the same conditions set out in Chater et al. (2016). **(A)** Mature brown intact Villersexel K3 wild-type sporophyte capsule attached to parental gametophyte. **(B)** A stacked and flattened image of a sporophyte capsule base of panel **(A)** dissected and face upward. **(C)** Close-up of stomata from sample in panel **(B)** illustrating darkening beneath the cavity. **(D)** Same capsule as in panels **(A–C)** mounted upside down to observe underlying sub-stomatal cavity. The darkening can be seen lining the sub-stomatal cavity which extends from the open pore into the base of capsule. **(E)** Ruptured brown Villersexel K3 sporophyte capsule attached to parental gametophyte. **(F)** A stacked and flattened image of the sporophyte capsule base in panel **(E)** dissected and faced upward. **(G)** Close-up of stomata from panel **(F)** illustrating darkening beneath the cavity. **(H)** The same capsule as in panels **(E–G)** mounted upside down. Darkening can again be seen lining large portions of the cavities which underlie the stomata. **(I)** Mature brown intact ppsmf1 sporophyte capsule attached to the parental gametophyte. **(J)** A stacked and flattened image of the sporophyte capsule base in panel **(I)** dissected and face upward. **(K)** Close-up of the astomate epidermis of *ppsmf1*. **(L)** The same capsule as in panesl **(I–K)** mounted upside down with no sub-stomatal cavities. **(M)** Ruptured *ppsmf1* sporophyte capsule attached to the parental gametophyte. **(N)** A stacked and flattened image of the sporophyte capsule base in panel **(M)** dissected and face upwards. **(O)** Close-up of the astomate epidermis of *ppsmf1*. **(P)** The same capsule as in panel **(O)** mounted upside down with no sub-stomatal cavities. Scales bars are as follows: **(A,E,I,M)** = 500 μm; **(B,F,J,N)** = 100 μm; **(C,D,G,H,K,L,O,P)** = 25 μm.

*Physcomitrium patens* subspecies *patens* (Hedwig) Mitten (Medina et al., 2019; Rensing et al., 2020) wild-type strains Gransden 2004, Gransden D12 and Villersexel, and previously published mutants were grown under sterile conditions on 42 mm Jiffy 7 peat pellets (Amazon, London). To produce data in Figures 2–7, pellets were first rehydrated using 40 ml of distilled water inside Magenta GA-7 culture vessels (Sigma-Aldrich, Gillingham, United Kingdom), sealed with Micropore tape (3 M, Maplewood, Minnesota, United States) and sterilized. Post-sterilization, a further 70 ml of sterilized distilled water was added. To produce sterile protonemal homogenate, a 1 × 9 cm plate of 6–10-day old BCDAT-grown tissue (Cove et al., 2009) was scraped from a cellophane disk (AA Packaging, Preston, United Kingdom) and placed in 15 ml of sterilized distilled water and homogenized for 20 s using a Polytron PT1200 (KINEMATICA AG, Luzern, Switzerland). Peat pellets were inoculated with either 1.5 ml of protonemal homogenate (Figures 2–6) or a 1.5 cm^2^ piece of tissue derived from 3-week-old BCDAT-grown tissue (Figure 1). To produce the sporophytes presented in Figure 8, plants were grown on agar plates (12 g l^–1^) supplemented with Knop medium (Egener et al., 2002; Frank et al., 2005) as previously described Chater et al. (2016).

### Growth Conditions

Plants were grown at 25°C under continuous light (140 μmol m^–2^ s^–1^ irradiance) for 8 to 12 weeks until large gametophores were produced. To induce gametangia, plants were moved to a Medicool MPR-161D(H) cabinet (Sanyo, Osaka, Japan) fitted with Phillips Master TL-D 90 De Luxe 18W/965 fluorescent lamps (Amsterdam, Netherlands) set to 18°C, 10 h light (100 μmol m^–2^ s^–1^ irradiance) and 15°C, 14 h dark. After 2–3 weeks, 40 ml of sterile distilled water was poured over plants to fertilize archegonia. For analysis of stomata on dry or wet grown sporophyte capsules, the following procedures were undertaken. For nascent sporophytes, samples were collected approximately 3 weeks after water application. For expanded green-to-yellow spore sporophytes, collection occurred approximately 5 weeks after water application. For browning sporophytes, samples were collected at approximately 6.5 weeks. For stomatal counts of browned sporophytes, samples were fixed in modified Carnoy’s solution (2:1 Ethanol: Glacial acetic acid) for 1 week prior to analysis. To identify dry grown sporophytes for Figure 7, capsules were located on peat pellets from lower-humidity zones of the moss canopy, identified by water repellent gametophores within the colony. Dry-and wet-grown samples were collected from similar heights and depths within colonies to minimize edge effects and micro-environments. We did not follow individual dry-grown sporophytes on individuals from fertilization until maturity. Plants cultivated on plates for results in Figure 8 were grown at 23°C, 16 h light, 8 h dark prior to sporophyte induction as in Hohe et al. (2002).

### Sample Preparation, Microscopy and Image Processing

For bright-field and fluorescence microscopy, spore capsules were excised from moss colonies and dissected in water. Imaging was performed on an Olympus BX-51 microscope fitted with Olympus DP71 camera (Tokyo, Japan). For fluorescence imaging of untreated samples, an Olympus U-RFL-T-200 UV lamp (Tokyo, Japan) with an LP 400 nm emission filter was used. To produce stacked images, multiple fields of view of a subject were obtained. Images were stacked using ImageJ (Schneider et al., 2012) and then flattened using the Z project function using either the Min Intensity or Max Intensity settings to compile flattened images. The moss colony image was taken using a Canon EOS 500D camera (Tokyo, Japan).

### DPBA Staining and Imaging

Mature browning capsules were fixed in Carnoy’s, and incubated for 1 h in either Diphenylboric acid-2-aminoethyl ester (DPBA; Sigma-Aldrich, Gillingham, United Kingdom) solution (0.25% DPBA, 0.02% Triton X-100 (v/v) or a control solution of 0.02% Triton X-100 solution (v/v). Dissected capsules were then imaged using a pE-2 UV Fluorescence Light Source (CoolLED, Andover, United Kingdom) on an Olympus BX-51 microscope. To excite samples 400 nm wavelength was utilized. To capture fluorescence at the appropriate wavelength a 455 nm emission filter was used. Bright-field and UV images of both the stained and treated controls were obtained using the same exposure settings.

### Statistical Analysis and Graphing

For comparisons of stomatal frequency, the total number of stomata were counted from 5 expanding and 5 expanded spore capsules and analyzed using a Student’s *t*–test. A stoma was classified as a GC with an obvious central pore. Dot-plot graphs were produced in R using the ggplot2 data visualization package (Wickham, 2009; R Development Core Team, 2012).

## Results

### Stomatal Development on the Sporophyte of *P. patens*

We observed the development of stomata in *P. patens*, which in common with other mosses, produces stomata only on the spore capsule of the sporophyte and not on the gametophyte (Paton and Pearce, 1957; Field et al., 2015; Caine et al., 2016; Chater et al., 2016; Merced and Renzaglia, 2017; Figure 2). Sporophyte development begins when a gametophytic egg cell is fertilized by a gametophyte sperm cell either via self-fertilization or from another individual (Perroud et al., 2011, 2019; Hiss et al., 2017). Post-fertilization, the diploid zygote divides asymmetrically to form a sporophyte consisting of an apical cell and basal cell (Sakakibara et al., 2008). The apical meristem derived from the apical cell is responsible for the development of the spore capsule on which stomata will form. The basal cell gives rise to the haustorium that anchors the sporophyte in the parent gametophore. An intercalary meristem implements further differentiation by producing a seta, a stalk containing conducting tissue which is responsible for elevating the developing sporophyte above the confines of the parent gametophore (Figures 2B,b).

As sporophyte development and expansion continues, seta development slows (Figure 2C). The calyptra, a gametophyte-derived protective cap (visible in Figures 2A,B) normally sits atop the sporophyte, and is ordinarily retained until just prior to sporophyte capsule maturity (Hiss et al., 2017). As the sporangium and stomatal regions continue to develop, the spore sac and stomatal lineage cells become increasing visible (Figures 2D,d). Within the spore sac, spores gradually develop (Wallace et al., 2015), and the capsule gradually matures leading to the sporophyte changing color from green to yellow, then orange, before finally browning (Chater et al., 2016; Hiss et al., 2017). Stomata also change color as the capsule matures, starting a relatively translucent color and gradually following the same color changes associated with the maturing sporophyte (Figures 2c–g). Following maturation, brown capsules dehisce through irregular lysis of epidermal cells, leading to rupture and spore dispersal; at this late stage of sporophyte development stomata often appeared to be “plugged” (Figure 2H).

### Quantifying Stomatal Development and Assessing Stomata on the Mature Sporophyte Epidermis

To investigate the timing of stomatal formation during *P. patens* wild-type sporophyte development we compared the number of stomata on expanding capsules (between the stages defined in Figures 2D,E) with the final stomatal number on fully expanded mature brown sporophytes Figure 2G (Figure 3). During expansion, the number of stomata was just over half that on fully mature spore capsules (Figures 3A–C). This indicates that stomatal development continues as sporophytes expand. At the partially expanded stage, all the stomata observed were spaced, with no clustering, but on mature capsules, small clusters of stomata were occasionally observed (Figures 3D–F). Although pairs, and very occasionally triplets of contiguously clustered stomata were observed on mature sporophytes, the majority (85–90%) were separated by at least one epidermal cell (Figure 3F). In addition to the occasional clusters, small patches devoid of stomata also infrequently occurred (Figure 3G), although these were not as large as the stomata-less zones previously observed in *pptmm* lines (Caine et al., 2016). On occasion, stomatal precursor cells with the characteristic GMC oval shape were observed in the mature sporophyte (Figures 3H–J), bearing similarity to arrested GMCs observed in other land plant lineages (Zhao and Sack, 1999; Pressel et al., 2014). During this study we used Gransden 2004, Gransden D12 or Villersexel wild-type *P. patens* strains. We did not observe any obvious differences in stomatal development or patterning, and our previous work (Chater et al., 2016), revealed no differences in stomatal number between the different backgrounds.

Many mature guard cells acquired an orangey hue prior to full browning of the sporophyte. To ascertain the chemical nature of the coloration we stained capsules with diphenylboric acid 2-amino ethyl ester (DPBA), which fluoresces under UV light in conjugation with flavonoid derivatives (Figures 3K–N). A substantial fluorescent signal was detected in the mature guard cells of stained capsules relative to unstained controls indicating the presence of flavonoids (Figure 3L). For controls, fluorescence was only detectable in the central pore regions and not in the guard cells (Figure 3N).

### Deciphering *P. patens* Stomatal Ontogeny

During the early stages of development, the unexpanded sporophyte was completely enclosed in a humid microenvironment provided by the calyptra (Figure 2B; Budke et al., 2011, 2012; Hiss et al., 2017). To ascertain whether stomatal development is initiated at this early developmental stage, the calyptra of young sporophytes was removed and the underlying epidermis was checked for the presence or absence of stomatal lineage cells (Figures 4A,B). At this developmental stage, no obvious stomatal lineage cells were detected using bright-field or fluorescence microscopy. The first developmental stage where stomata lineage cells were clearly apparent (Figures 4C,D), was on sporophytes equivalent to those in Figure 2C. As the young sporangium expanded further (Figure 2D), in addition to maturing guard cells with fluorescent cuticular pores (Figure 4D), other stomatal lineage cells were also present (Figures 4E–L). These included distinctively circular unexpanded cells that had radiating epidermal cells surrounding them (Figure 4E). Based on their orientation to other cells and lack of expansion, the circular cells were probably GMC precursors. Although we observed lots of variation in epidermal cell size and orientation, we did not identify asymmetric entry divisions equivalent to those described in the early stomatal lineage of vascular land plants (Zhao and Sack, 1999), in agreement with previous reports that such divisions are absent during the very earliest stages of moss stomatal development (Vaten and Bergmann, 2012).

Using our combined bright-field and fluorescence microscopy technique, we characterized the developmental changes that take place once a GMC is formed and subsequently then undergoes an incomplete division to form a GC (Figures 4E–N). For clarity, we defined pore opening, rather than cell plate formation, as the point when a GC formed in *P. patens*. This characterization is in line with the designation of guard cells in Arabidopsis by Zhao and Sack (1999). We detected a number of expanded oval shaped putative GMCs on the expanding sporophyte (Figures 4F–H) in addition to the probable GMC precursors discussed above (Figure 4E, also marked by blue dots in the epidermal and sub-epidermal images shown in Figures 4I–L). These cell conformations included; cells with prominent, radially aligned circular bodies consisting of chloroplasts and starch granules containing a centralized cell plate (Figure 4F and marked by green dots in Figures 4I–L); cells with sparse, fragmented cellular matter containing a central cell plate (Figure 4G); and cells with densely fragmented cellular matter (Figure 4H, and marked by yellow dots in Figures 4I–L and Supplementary Figure S1). Using fluorescence imaging we observed stomatal pores in oval cells with fragmented cellular matter (yellow dots), but not in cells with circular organelles (green dots, early GMCs) or those with sparse fragmented cellular matter (late GMCs, Figures 4I–L, 5). The composition of the observed fluorescent material is not known, but possibilities include cutin or wax deposition in the pore wall and/or pectin/mucilage build-up (Lee and Priestley, 1924; Isaac, 1941; Smith, 1955; Sack and Paolillo, 1983; Pressel et al., 2014; Merced and Renzaglia, 2017). Once incomplete symmetric division of a GMC was finished, the cellular contents of GCs re-aggregated (Figures 4M,N and Supplementary Figure S1). These data suggest that enlarged GMCs (early GMCs) first develop with aggregated circular organelles and/or starch granules which then dissipate in late GMCs, before fragmentation of contents occurs throughout the cell and pore formation occurs which marks the differentiation from GMC to GC (Figure 4O). The formation of the GC results in the re-assembly of the cell contents and gradual browning and flavonoid formation as the stoma matures.

Whilst observing stomatal development, we noticed meristematic activity in early GMCs (Figure 5). This was in contrast to previous reports in which non-vascular land plant GMCs always divide or differentiate directly to form a GC or pair of GCs (Sack and Paolillo, 1985; Pressel et al., 2014; Merced and Renzaglia, 2016). We found that when two *P. patens* early GMCs formed adjacently, one of the GMCs had the potential to undergo a GMC spacing division resulting in the formation of an intervening spacer cell (SPC) (Figure 5). During a spacing division, aggregated cellular organelles in the dividing GMC were at first spread throughout the dividing cell (see right GMC in Figures 5A,B). As the division occurred over a 2 h-period, the organelles dissipated and migrated to the most distal part of the dividing GMC as it moved away from the previously adjacent GMC (Figures 5C–H). Meanwhile over the same period, in the stationary GMC the aggregated organelles dissipated (see left GMC in Figures 5C–H). Autofluorescence profiles of the division, taken over the 2 h period, suggest that both cells start at the early GMC stage, and at no point does pore formation begin to occur (Figures 5B,D,H). These data indicate that *P. patens* GMCs can undergo spacing divisions which would enable more dynamic control over stomatal patterning and development than previously thought. It remains unclear whether renewed GMCs could undergo further meristematic activity by undergoing additional GMC spacing divisions as it was not possible to continue tracking the live dissected samples over longer periods (Figure 5L). Similarly, it remains unknown whether the SPC produced from a GMC spacing division could maintain or revert to GMC identity and become a stoma, if spacing permitted.

### Identifying Controllers of Cell Fate and Polarity: Stomatal Development Ontogeny in *P. patens* Development and Patterning Mutants

To further understand *P. patens* stomatal development and spacing processes at the molecular level, a reverse genetics approach was taken and the development of stomatal mutants generated in previous studies were studied (Caine et al., 2016; Chater et al., 2016). Observations were taken at equivalent developmental stages to the wild-type sporophytes in Figures 4, 5. Both *ppsmf1* and *ppscrm1* single deletion mutants failed to form early GMCs (Figures 6A–H). We observed some small cells in these lines, however, it was not possible to conclude whether these were pre-cursors to GMCs or nascent epidermal cells. Observations of *ppepf1* sporophytes revealed contiguous clustering of early and more advanced GMCs which were irregularly orientated, possibly due to an inability to undergo GMC spacing divisions (Figures 6K,N). Conversely, over-expression of *PpEPF1* produced early GMCs with ectopic organelle formation indicative of endoreduplication, or alternatively large areas without any early GMCs (Figures 6O,P). Taking *ppepf1* and *PpEPF1OE* phenotypes together, it appears that PpEPF1 prevents cells adjacent to early GMCs from assuming early GMC identity. Moreover, PpEPF1 appears to govern early GMC spacing divisions by regulating GMC duplication, and also assists in setting the correct orientation of division (Figures 6K–P).

Mature *pptmm* mutant capsules exhibit a range of stomatal patterning phenotypes which vary both between and within individual sporophytes and between sporophytes (Caine et al., 2016). *pptmm* phenotypes were tracked during early sporophyte development (Figures 6Q–X). In the young *pptmm* epidermis, there were zones devoid of stomatal precursors (Figures 6Q,R), irregularly small GMCs (Figures 6S,T), early GMCs dividing toward each other (Figures 6U,V), and areas with clustered early GMCs with irregular cellular orientations (Figures 6W,X). Overall, these phenotypes suggest that PpTMM is required for the correct regulation of early GMCs in a number of ways. PpTMM enhanced entry into the stomatal lineage by promoting early GMC formation when no other GMCs were present (Figures 6Q–T) and once early GMCs had formed, PpTMM acted to prevent excessive ectopic spacing divisions of early GMCs (Figures 6U,V). PpTMM also specified the orientation of GMC divisions and regulated early GMC identity in neighboring cells, thereby preventing clustering (Figures 6U–X).

### Growth in Wet or Dry Conditions Influences the Fate of Mature *P. patens* Stomata

As sporophyte capsules expanded (Figures 2C–F), guard cells continued to develop, and once formed generated a pore linking the sub-stomatal cavity with the surrounding environment (Figure 7). We observed that when sporophytes matured under wet conditions, GC pores were often occluded. When viewed under UV light these plugged pores displayed an enhanced autofluorescent haze, probably due to increased secretion of mucilage (Figures 7A,B). Conversely, sporophytes which matured under drier conditions (Figures 7C,D) had stomata that typically displayed autofluorescence from the inner walls of the GC pore and inner cavity (compare Figure 7D to Figure 7B). In some instances, the sub-stomatal area become darkened, perhaps due to dried mucilage (Figure 7E), and this was evident even from a distance (Figure 7F). The stomata and underlying cavities of fully expanded green-to-yellow capsules often had plugged pores when grown under wet conditions, and open pores with darkened cavities were frequently detectable in capsules grown under dry conditions (Figures 7G,K,I,M). Typically, the autofluorescence signals from “wet-grown” capsule stomata arose from the clogged pore, whereas dry-grown capsules emitted auto-fluorescence from the underlying sub-stomatal cavities where mucilage had receded (compare Figures 7H,L with Figures 7J,N).

To ascertain whether the darkening effect attributed to drying mucilage also occurred in *ppsmf1*, which fails to produce stomata or sub-stomatal cavities (Chater et al., 2016), we examined dry-grown mature capsules prior to- and after- sporophyte rupture (Figure 8). As expected, *ppsmf1* presented no darkening in either mature intact or ruptured sporophytes (Compare Figures 8A–H with Figures 8I–P). This observation suggests that stomata are essential for the induction of internal darkening during capsule dry-down in *P. patens*. These observations help to explain the delayed sporophyte rupture of *ppsmf1* mutants (Chater et al., 2016) and provide further evidence that bryophyte stomata play an important role in capsule drying and spore dispersal (Duckett et al., 2009; Pressel et al., 2014; Chater et al., 2016; Merced and Renzaglia, 2017).

## Discussion

### Building Increasingly Robust Stomatal Developmental Modules

**FIGURE 9:**
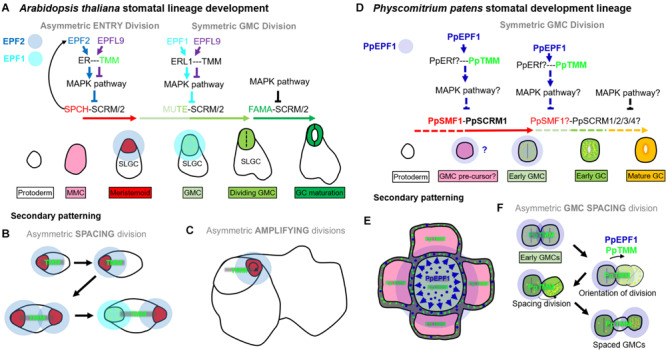
Contrasting stomatal development in *Arabidopsis thaliana* and *Physcomitrium patens*. In Arabidopsis, asymmetric entry **(A)**, spacing **(B)**, and amplifying **(C)** divisions are regulated by SPEECHLESS (SPCH) and ICE1/SCREAM (SCRM) or SCRM2. In all three types of divisions, SPCH activity is under phospho-regulatory control, and this occurs via the MAPK pathway. The MAPK pathway facilitates signals from the plasma membrane exterior to be transduced into the nucleus. ERECTA family proteins [particularly ERECTA (ER)], modulated by TOO MANY MOUTHS (TMM) convey such signals. If the EPIDERMAL PATTERNING FACTOR (EPF), EPF2 successfully binds to ER then stomatal lineage entry is inhibited. If the EPF-like (EPFL) peptide, EPFL9 binds, the MAPK pathway activity is reduced, allowing the SPCH-SCRM/2 heterodimers to initiate asymmetric divisions from meristemoid mother cells (MMC), leading to new meristemoid formation [see red cell in entry **(A)** or spacing **(B)** division], or the renewal of an existing meristemoid [see red cell in amplifying **(C)** divisions]. SPCH also upregulates EPF2 and thus regulates its own activity through negative feedback. Later MUTE, together with SCRM/2, orchestrates the advancement of the meristemoid to GMC, and then participates in the GMC division. EPF1 inhibits MUTE activity and EPFL9 promotes it, again via elements of a MAPK pathway. What drives EPF1 expression is still unclear. Lastly, FAMA together with SCRM/2 permits correct GC identity and this stage is also governed by various elements of the MAPK pathway. Typically EPF2 is secreted from meristemoids during early stomatal development, primarily to restrict SPCH activity in neighboring stomatal lineage cells [see translucent blue circles around meristemoids in panels **(A–C)**]. EPF1 is secreted later during the meristemoid to GMC transition, and continuing into GMC division [see translucent light blue circles around GMCs in panels **(A,B)**]. **(D)** PpSMF1 and PpSCRM1 both facilitate stomatal lineage advancement to an early GMC. This may also include promoting early GMC pre-cursor fate. PpTMM promotes the initial formation of GMCs, and PpEPF1 may also be involved by preventing initial cells from obtaining early GMC fate. These signals are probably conveyed by a number of PpERECTA proteins (Caine et al., 2016) and a MAPK pathway. PpEPF1 function could normally take place once an initial GMC has been specified, although further work is required to establish whether this is the case. Downstream of the early GMC formation it is unclear as to what, if any, roles PpSMF1 and/or PpSCRM1/2/3/4 undertake. All lines which are dashed are hypothesized mechanisms as are the functions of proteins which are followed by question marks. **(E)** Secondary patterning occurs when early GMC cells may secrete PpEPF1 (blue circles) to signal to nearby early GMC pre-cursor. PpTMM, on the early GMC pre-cursor (green sticks), probably transduces PpEPF1 signal and thereby preventing adjacent early GMCs from forming. **(F)** GMC asymmetric spacing divisions occur when two early GMCs concurrently arise next to each other, maybe from cells which concurrently differentiate from GMC pre-cursor origin. PpEPF1 may be secreted by both early GMCs, and via PpTMM signaling assists in the orientation of division so that GMCs are not touching. The spacing division can then unfold and cellular organelles move in a polar fashion to the distal part of the dividing cell. This will eventually lead to GMCs which are separated via a spacer cell (SPC). PpEPF1 presence with PpTMM ensures these processes occur in the correct orientation. It is unclear whether the new SC has the capability to re-enter the stomatal lineage.

In Arabidopsis, as in most other land plants, stomata are spaced by at least one intervening epidermal pavement cell (Hara et al., 2007; Rudall et al., 2013; Caine et al., 2016). This is achieved through orientated amplifying and spacing divisions of stomatal precursors, so that stomatal lineage cells are typically bordered by SLGCs or epidermal pavement cells (Geisler et al., 1998, 2000; Zhao and Sack, 1999; Figures 9A–C). EPF2 negatively regulates SPCH-SCRM/2 activity in the early stages of the stomatal lineage; EPF1 negatively regulates MUTE-SCRM/2 as meristemoids transition to GMCs; and TMM is required for EPF signal transduction (Hara et al., 2007; Hunt and Gray, 2009; Qi et al., 2017). Our observations indicate that mosses also influence stomatal patterning via orientated asymmetric cell divisions, albeit at the early GMC stage, and this is regulated by a similar molecular signaling pathway: The one moss EPF (PpEPF1) and RLP (PpTMM) are both required for correct stomatal spacing (Figures 4, 5, 6I–X, 9D–F). PpSMF1 and PpSCRM1 are also necessary, as without these transcription factors there is no GMC formation.

A key difference between the Arabidopsis asymmetric divisions and the observed *P. patens* spacing division mechanism (Figures 5, 6) is that Arabidopsis has the ability to undergo such divisions prior to GMC formation; a stage which is characterized in both species by small cells with aggregated chloroplasts (Lucas et al., 2006; Figures 4, 9). This post-ponement of the GMC state in angiosperms may have been made possible by the duplication(s) of members of both the *SMF* and *EPF* gene families. In particular, the evolution of *EPF2* and *SPCH* genes enabled earlier temporal control of asymmetric divisions (compare Figures 9A–C with Figures 9D,F), which led to greater developmental plasticity and more accurately spaced stomata. In some respects, PpEPF1 behaves similarly, to both EPF2 and EPF1 in that it functions to prevent neighboring cells of early GMCs, such as GMC pre-cursors, from becoming early GMCs. This is similar to how both EPF2 and EPF1 regulate Arabidopsis MMCs and SLGCs which neighbour meristemoids and GMCs (compare Figures 9B,E). On the other hand, PpEPF1 also shows similarity with EPF2 functionality during amplifying divisions to maintain cell fate (Figures 9C,F). This is because, as highlighted, although ectopic early GMC divisions occur in *ppepf1* plants (see Figures 5M,N), instead of terminating as small cells as in *epf2* plants, the early GMCs of *ppepf1* carry on to become stomata and, hence, contiguous clustering occurs (Hara et al., 2009; Hunt and Gray, 2009; Caine et al., 2016).

Whilst we show PpSMF1 and PpSCRM1 regulate early GMC formation (Figures 6A–H), we are unable to confirm that these proteins also play a part in GMC precursor formation and the GMC symmetric division leading to GC formation. Interestingly, the E-box DNA binding domain of FAMA, which is integral for GC formation in Arabidopsis, is also present in PpSMF1 (Chater et al., 2016), and complementation studies in both Arabidopsis *mute* and *fama* have shown that PpSMF1 can partially rescue both mutant lines (MacAlister and Bergmann, 2011). Further functional motif studies are required to understand the ancestral roles of SMF and SCRM bHLHs, and their divergence and specialization across stomatal evolution.

Our data suggest that a more complex form of stomatal patterning exists in *P. patens* than was previously thought. We propose that moss GMCs have the capacity to alter their cell fate, rather than directly transitioning to GCs they may instead undergo asymmetric spacing divisions. This differs from the situation reported in the closely related *Funaria hygrometrica*, where stomata are exclusively spaced via divisions of close-by epidermal cells (Merced and Renzaglia, 2016). Our analysis of *ppsmf1* and *ppscrm1* capsules illustrates that GMCs only form when both of these key bHLH genes are present; no early GMCs were found in either of the mutant backgrounds throughout sporophyte development. This confirms that the early GMCs involved with *P. patens* spacing divisions (Figure 5) are indeed GMCs, and not undifferentiated epidermal cells that integrate correct stomatal patterning post-GC formation, as described in *F. hygrometrica.*

For ancient sporophytes, the evolution of a GMC spacing division would have permitted a more refined regulation of stomatal development. Later, as plant lineages evolved, regulatory control may have been modified to enable asymmetric entry divisions prior to the formation of the GMC. This would have enabled stomatal development to be corrected at an earlier time-point, thereby further optimizing stomatal placement. At the molecular level this is particularly evident in *SPCH* evolution, as this gene (and encoded protein) has many regulatory points that govern if and when stomatal development is initiated (Lampard, 2009; Sugano et al., 2010; Gudesblat et al., 2012; Tricker et al., 2012; Chater et al., 2017; Lau et al., 2018). As photosynthetic capacity became more important to increasingly large sporophytes, perhaps *EPFL9*-type genes evolved to enable signals from the mesophyll to be more tightly integrated into the stomatal development module.

### Further Considerations for Stomatal Function in *P. patens*

It has been proposed that stomata are “monophyletic” structures across land plants (Raven, 2002; Caine et al., 2016). However, the possibility of convergent evolution of stomata across plant groups has also been argued (Raven, 2002; Pressel et al., 2014). Recent molecular and physiological analyses suggest that the mechanisms of stomatal function and development are broadly conserved across land plants (Chater et al., 2011, 2016; Ruszala et al., 2011; Doi et al., 2015; Lind et al., 2015; Caine et al., 2016; Harris et al., 2020). However, in earlier diverging lineages including the bryophytes, the divergent physiology and functions of stomata continue to be debated (Duckett et al., 2009; Chater et al., 2011, 2013, 2016; Pressel et al., 2014; Field et al., 2015; Chen et al., 2016; McAdam and Brodribb, 2016; Hõrak et al., 2017; Grantz et al., 2019). While this matter requires further study, it has become clear that for bryophytes stomata are important in aiding sporophyte drying and subsequent rupture for dehiscence, leading to spore dispersal (Duckett et al., 2009; Merced and Renzaglia, 2013; Chater et al., 2016), and our anatomical observations here further support this.

In addition to aiding capsule drying, it has been suggested that the positioning of stomata around the base of moss spore capsules may also aid in water and nutrient uptake from the parent gametophyte (Haig, 2013). Moreover, owing to stomata being positioned above spongy tissues, it has been further suggested that they may be important in permitting gas exchange for photosynthesis (Merced and Renzaglia, 2013). Our observations suggest that the function of moss stomata might also vary depending on the environment in which a given capsule develops, and this is related to whether stomata become occluded or not. In wet environments, stomata readily become plugged with auto-fluorescent cuticular material that perhaps prevents water and/or pathogens entering sub-stomatal cavities (Figure 7). Whether this inhibits gaseous exchange and impacts on matrotrophy is unknown. Conversely, in drier habitats, stomata are often open as the sporophyte capsule enlarges, perhaps enhancing expanding sporophyte gas exchange and water and nutrient acquisition from the parental gametophore (Figures 7, 8). We suggest that the darkening of drying capsules is a result of receding mucilage and related to the material observed in hornwort stomata that may assist in capsule rupture (Pressel et al., 2014). Our data support this assumption, as *ppsmf1* capsules lacking stomata and their cavities demonstrate delayed rupture (Chater et al., 2016) and do not undergo sub-epidermal darkening (Figure 8). Taken together, these observations suggest that bryophyte stomata, like the stomata of vascular plants, permit the controlled release of water, and thereby enhance the efficiency of mature capsule drying and rupture, and increasing the distance of spore dispersal. For *P. patens*, an ephemeral moss of riparian habitats, this may translate into the improved fitness of an individual by increasing the probability for spores to reach aqueous environments such as rivers and lakes.

## Data Availability Statement

The raw data supporting the conclusions of this article will be made available by the authors, without undue reservation, to any qualified researcher.

## Author Contributions

RC performed the experiments. All authors interpreted the data and wrote the manuscript. AF and JG conceived the project.

## Conflict of Interest

The authors declare that the research was conducted in the absence of any commercial or financial relationships that could be construed as a potential conflict of interest.
