# Supplementary material for: Stomata and Sporophytes of the Model Moss Physcomitrium patens
Source: Front Plant Sci. 2020 May 25;11:643. doi: 10.3389/fpls.2020.00643 (PMC7261847; doi:10.3389/fpls.2020.00643)
Supplement: Supplementary file 1 [file Image_1.pdf]

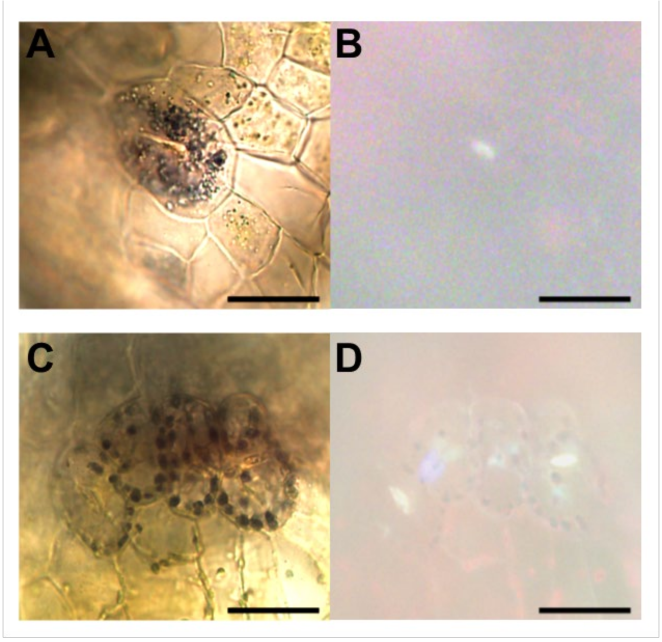

Supplementary Figure 1. Starch presence in stomatal lineage cells at both the early developing GC stages. (A) Wild-type Gransden D12 bright-field image of a dividing early GC stained with Lugol solution. The fragmented organelles have a blue colour indicating starch presence (B) Equivalent fluorescence image illustrating early pore formation of the dividing Early GC. (C) Bright-field image of developing GCs indicating the presence of starch, this time within aggregated organelles around the cell periphery in *ppepf1*. (D) Equivalent fluorescent image highlighting pore formation in the nascent stomata. Scale bar = 20µm.
